# Identification of Genes Regulated by the Antitermination Factor NasT during Denitrification in *Bradyrhizobium diazoefficiens*

**DOI:** 10.1264/jsme2.ME19033

**Published:** 2019-06-28

**Authors:** Cristina Sánchez, Arthur Fernandes Siqueira, Hisayuki Mitsui, Kiwamu Minamisawa

**Affiliations:** 1 Graduate School of Life Sciences, Tohoku University 2–1–1 Katahira, Aoba-ku, Sendai 980–8577 Japan

**Keywords:** *Bradyrhizobium*, denitrification, NasT, transcriptional regulation, RNA-seq

## Abstract

The soybean symbiont *Bradyrhizobium diazoefficiens* grows anaerobically in the presence of nitrate using the denitrification pathway, which involves the *nap*, *nir*, *nor*, and *nos* genes. We previously showed that NasT acts as a transcription antitermination regulator for *nap* and *nos* gene expression. In the present study, we investigated the targets of NasT in *B. diazoefficiens* during denitrifying growth by performing transcription profiling with RNA-seq and quantitative reverse-transcription PCR. Most of the genes with altered expression in the absence of NasT were related to nitrogen metabolism, specifically several systems for branched-chain amino acid transport. The present results suggest that the reduced expression of genes involved in nitrogen acquisition leads to the induction of alternative sets of genes with similar functions. The Δ*nasT* mutant of *B. diazoefficiens* grew better than the wild type under denitrifying conditions. However, this enhanced growth was completely abolished by an additional loss of the *narK* or *bjgb* genes, which encode cytoplasmic systems for nitrite and nitric oxide detoxification, respectively. Since the expression of *narK* and *bjgb* was increased in the Δ*nasT* mutant, the growth of the Δ*nasT* mutant may be promoted by increased detoxification activity.

Soybean hosts the N_2_-fixing bacterium *Bradyrhizobium diazoefficiens* (reclassified from *B. japonicum* [3]), which has the ability to grow under low oxygen conditions by sequentially reducing nitrate (NO_3_^−^) to N_2_ via the denitrification pathway. Denitrification is a dissimilatory pathway that requires four enzymes in *B. diazoefficiens*: periplasmic nitrate reductase (Nap), nitrite (NO_2_^−^) reductase (NirK), nitric oxide (NO) reductase (cNor), and nitrous oxide (N_2_O) reductase (Nos). These enzymes and their accessory functions are encoded by the *napEDABC*, *nirKV*, *norCBQD*, and *nosRZDYFLX* gene clusters, respectively (28). Denitrification may be advantageous for bradyrhizobial cell survival in the soybean rhizosphere and for root colonization after oxygen depletion (18). Under symbiotic conditions, denitrification contributes to the production and detoxification of NO, an important signaling molecule for the establishment and functioning of symbiosis (27). Additionally, bradyrhizobial denitrification is involved in both the production and mitigation of the greenhouse gas N_2_O in soybean fields (9, 11).

The *nasST* operon encodes a NO_3_^−^ and NO_2_^−^ sensor/transcriptional antitermination regulatory system. This system was initially considered to be involved in the NO_3_^−^/NO_2_^−^-responsive regulation of *nas* genes for the NO_3_^−^ assimilation pathway in bacteria, including *B. diazoefficiens* (1, 15, 16, 25, 38). Since *nasST* genes are located separately from the NO_3_^−^/NO_2_^−^ assimilation gene cluster in some bacteria, a possible role for the regulation of other metabolic pathways was suggested (15). We showed that the expression of *nap and nos* genes was weaker under anaerobic NO_3_^−^ respiration conditions (hereafter ‘denitrifying conditions’) in the *B. diazoefficiens* Δ*nasT* mutant than in the wild type (30). Other targets of this regulatory system are currently unknown in this bacterium.

NasS and NasT form a complex that dissociates when NasS senses NO_3_^−^ at a micromolar concentration (8, 16, 30). NasT is an ANTAR (AmiR and NasR transcription antitermination regulator) family protein (34). When it is released from NasS, this protein interacts directly with the 5′-leader region of *nosR* mRNA and interferes with the formation of a terminator structure, allowing for the read-through transcription of *nos* genes (31). A similar antitermination mechanism is expected for other targets of NasT, as suggested for the regulation of *nas* genes in *B. diazoefficiens* and other bacteria (1, 16, 25, 38). The Δ*nasT* mutant has been shown to grow better than the wild type under denitrifying conditions (30); this was an unexpected observation because the growth of *B. diazoefficiens* is completely dependent on *nap* genes under denitrifying conditions (4).

The main objective of the present study was to investigate the targets of NasT in *B. diazoefficiens* under denitrifying conditions. We herein showed that NasT regulated a number of genes involved in nitrogen metabolism. We also characterized *B. diazoefficiens* mutants in different genes of the *nas* operon that are relevant to NO_2_^−^ and NO detoxification in the cytoplasm (*narK* and *bjgb*) and demonstrated the involvement of these genes in the enhanced growth of the Δ*nasT* mutant under denitrifying conditions.

## Materials and Methods

### Bacterial strains and growth conditions

The strains used in the present study are listed in Table 1. Cells of *B. diazoefficiens* were pre-cultured aerobically with reciprocal shaking (300 rpm, 30°C) in HM salt medium (HEPES; 1.3 g L^−1^; MES, 1.1 g L^−1^; Na_2_HPO_4_, 0.125 g L^−1^; Na_2_SO_4_, 0.25 g L^−1^; NH_4_Cl, 0.32 g L^−1^; MgSO_4_ 7H_2_O, 0.18 g L^−1^; FeCl_3_, 0.004 g L^−1^; CaCl_2_ 2H_2_O, 0.013 g L^−1^; pH 6.8) supplemented with 0.1% l-(+)-arabinose and 0.25% (w/v) yeast extract (2, 26). *Escherichia coli* cells were grown at 37°C in Luria–Bertani medium (20). The following antibiotics were used for *B. diazoefficiens*: kanamycin (Km; 100 μg mL^−1^), spectinomycin (Sp; 100 μg mL^−1^), streptomycin (100 μg mL^−1^), and polymyxin B (50 μg mL^−1^); and for *E. coli*: Km (50 μg mL^−1^) and Sp (50 μg mL^−1^).

In growth experiments under denitrifying conditions, cells were inoculated into 5 mL (optical density ~0.01 at 660 nm) of HM medium supplemented with trace metals (26) and 10 mM KNO_3_ (HMMN) in 35-mL tubes. The tubes were sealed with rubber stoppers and the gas phase was replaced with 100% N_2_ in a vacuum line (26, 35). Cells were grown at 30°C with reciprocal shaking at 300 rpm. Growth was measured daily by recording optical density at 660 nm. Extracellular NO_3_^−^ concentrations were measured as described previously (35).

### RNA isolation and sequencing

Cells of *B. diazoefficiens* were inoculated into 20 mL (optical density ~0.01 at 660 nm) of HMMN medium in 100-mL bottles and reciprocally shaken (100 rpm, 30°C) for 24 h under denitrifying conditions. The isolation of total RNA, the DNaseI treatment, and cDNA synthesis were performed as described previously (31 and references therein). Two biological replications were processed for each strain (wild-type USDA 110 and Δ*nasT* mutant). In each cDNA sample (four in total), 5 μg was used for the RNA sequencing analysis. The removal of ribosomal RNAs with the Ribo-Zero Magnetic Kit for Gram-negative Bacteria (Epicentre, Madison, WI, USA), cDNA library preparation with the Illumina TruSeq Stranded mRNA LT Sample Prep Kit (Illumina, San Diego, CA, USA), and sequencing on the Illumina HiSeq 2000 Sequencing System in the paired-end mode running 100×2 cycles were performed by Hokkaido System Science (http://www.hssnet.co.jp/). In each of the four samples, ~45 million reads were generated; ~83% of the reads had Q (Phred quality score) ≥30 for each of the four samples (Table S1).

### Bioinformatic analysis

Read trimming, the mapping of reads to the reference genome, read counting, normalization to RPKM (reads kilobase^−1^ million mapped reads^−1^), and calculations of expression values were performed with the CLC Genomics Workbench software 9.5.3. (https://www.qiagenbioinformatics.com/). Based on the total reads generated, approximately ~85% were trimmed, and from those, ~42% were mapped onto the *B. diazoefficiens* genome (GenBank accession number: NC_004463) (Table S1). Genes with fewer than 10 reads per 1 million mRNA reads were omitted from subsequent analyses. A gene with a fold change ≥2 or ≤−2 and a *q* value (estimate of the false discovery rate) ≤0.05 was considered to be up- or down-regulated, respectively. The Rhizobase (http://genome.annotation.jp/rhizobase) and KEGG (http://www.genome.jp/kegg/) databases were used for pathway analyses.

### RNA sequencing data accession number

RNA sequencing data have been deposited in the NCBI Gene Expression Omnibus and are accessible through GEO Series accession number GSE130301 (https://www.ncbi.nlm.nih.gov/geo/query/acc.cgi?acc=GSE130301).

### Validation of differential expression

To validate RNA sequencing results, quantitative reverse transcription-PCR (qRT-PCR) was performed on selected genes using a LightCycler Nano Instrument (Roche, Basel, Switzerland) with the FastStart Essential DNA Green Master (Roche) and specific primers for *sigA* (*sigA*f/*sigA*r), *nosR* (*nosR*f/*nosR*r), *nosZ* (*nosZ*f/*nosZ*r), *napA* (*napA*f/*napA*r), *nirK* (*nirK*f/*nirK*r), *norB* (*norB*f/*norB*r), *narK* (*narK*f/*narK*r), *nasC* (*nasC*f/*nasC*r), and bll3385 (bll3385f/bll3385r) (Table S2). The PCR program was set according to the manufacturer’s instructions. The specificity of PCR amplification was confirmed by a melting-curve analysis. This analysis was performed in duplicate for each of the two independent RNA samples. Expression levels calculated by the 2^−ΔΔCt^ method (33) were normalized to the *sigA* level and expressed relative to the values for the wild type.

### Construction of mutant strains

*B. diazoefficiens* Δ*narK*, Δ*bjgb*, and Δ*nasC* mutants were constructed by overlap extensions (10). In separate PCRs, two fragments (600–700 nucleotides each) of the target sequence were amplified using PrimeSTAR Max DNA Polymerase (TaKaRa Bio, Kusatsu, Japan) and the following primer sets: *narK*_01/*narK*_02 (to generate *narK*-A), *narK*_03/*narK*_04 (*narK*-B), *bjgb*_01/*bjgb*_02 (*bjgb*-A), *bjgb*_03/*bjgb*_04 (*bjgb*-B), *nasC*_01/*nasC*_02 (*nasC*-A), and *nasC*_03/*nasC*_04 (*nasC*-B). Primer sequences are listed in Table S2. *narK*-A and *narK*-B, *bjgb*-A and *bjgb*-B, and *nasC*-A and *nasC*-B fragments were then fused in second PCR with the same polymerase and the primer sets *narK*_01/*narK*_04, *bjgb*_01/*bgjb*_04, and *nasC*_01/*nasC*_04 (Table S2), respectively. PCR products were cloned as ~1.3-kb BamHI–PstI fragments for *narK*-AB and *bjgb*-AB and as ~1.3-kb EcoRI–BamHI fragments for *nasC*-AB into the pK18*mobsacB* vector (32). The resulting plasmids (pΔ*narK*, pΔ*bjgb*, and pΔ*nasC*; Table 1) were transferred by conjugation from *E. coli* DH5α to *B. diazoefficiens* using pRK2013 as a helper plasmid (5) to generate markerless deletions, as described previously (32). Kanamycin-resistant transconjugants were selected and grown in the presence of 10% sucrose to force the loss of the vector-encoded *sacB* gene. The resulting colonies were checked for Km sensitivity. The desired deletions were confirmed by PCR. To obtain Δ*napA*-Δ*nasT*, Δ*narK*-Δ*nasT*, Δ*bjgb*-Δ*nasT*, and Δ*nasC*-Δ*nasT* double mutants, the plasmid pΔ*nasT* (Table 1) was transferred by conjugation from *E. coli* DH5*α* to *B. diazoefficiens* Δ *napA* (Table 1), Δ*narK*, Δ*bjgb*, and Δ*nasC*, respectively, as described above. The desired deletions were confirmed by PCR using the primer set *nasT*_01/*nasT*_02 (Table S2).

## Results and Discussion

### The NasT regulon is mainly composed of genes involved in nitrogen metabolism

**Fig. 1:**
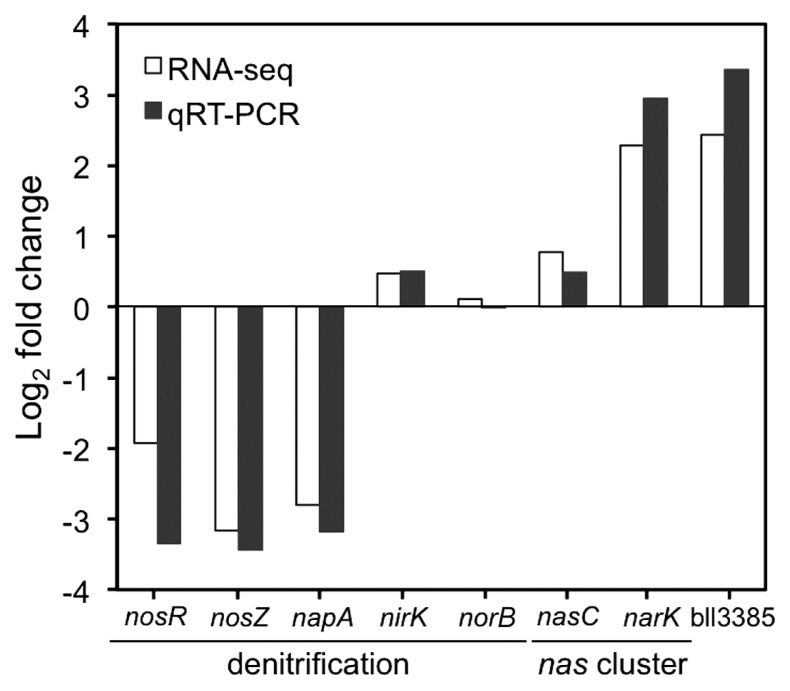
Comparison of logarithm-transformed expression data generated by RNA-seq (white bars) and qRT-PCR (black bars). Fold-change values refer to differences in expression when the *B. diazoefficiens* Δ*nasT* mutant was compared with wild-type USDA 110. Data are means of two independent RNA samples.

We used RNA-seq for the transcription profiling of the wild type and Δ*nasT* mutant, which were grown under anaerobic conditions in the presence of NO_3_^−^ at 10 mM as the electron acceptor with both ammonia and organic nitrogen as nitrogen sources (2, 26). Under these conditions, NasT proteins are expected to be fully active (8, 30). We found that 77 genes were differentially expressed in the Δ*nasT* mutant from those in the wild-type strain, with 40 genes being down-regulated and 37 genes being up-regulated in the Δ*nasT* mutant (Table 2 and 3). Consistent with previous studies (30, 31), the complete *napEDABC* (for dissimilatory NO_3_^−^ reductase) and *nosRZDFYLX* (for N_2_O reductase) gene clusters were decreased in the Δ*nasT* mutant (Table 2), whereas the expression of *nirKV* (for dissimilatory NO_2_^−^ reductase) and *norEBCQD* (for NO reductase) gene clusters remained unchanged (Fig. 1). This result was further validated using qRT-PCR for eight genes, including the denitrification genes *nosR*, *nosZ*, *napA*, *nirK*, and *norC* (Fig. 1).

In addition to denitrification genes, most of the genes with altered expression in the Δ*nasT* mutant were also related to nitrogen metabolism; a number of genes are putatively involved in the transport of amino acids, specifically, branched-chain amino acids (LIV, for L-leucine, L-isoleucine, and L-valine) (Table 2 and 3). This is in agreement with the observation that the majority of ANTAR-associated genes are related to nitrogen metabolism (24).

We found that some genes dedicated to similar processes varied in opposing directions in the Δ*nasT* mutant. In putative systems encoding LIV transport, blr6443/45-6447 and bll0913 were down-regulated, while the blr2922-2926 cluster and bll3383-3386 were up-regulated (Table 2 and 3). In addition, for aerobic carbon monoxide dehydrogenase, blr0335 (*coxS*) and blr0336 (*coxL*) were down-regulated, and bll3376 (*coxS*) and bll3377 (*coxM*) were up-regulated (Table 2 and 3).

The presence of alternative sets of LIV transport systems in *B. diazoefficiens* may be explained by LIV being important nutrients in bacterial physiology, with roles that range from supporting protein synthesis to signaling and fine-tuning the adaptation to amino acid starvation (12). Furthermore, LIV transport is essential for N_2_ fixation because symbiotic rhizobial cells become auxotrophs for LIV and depend on the plant for their supply (22, 23). The expression of bll3386, a transcriptional regulator that belongs to the bll3383-3386 gene cluster (Table 3), was induced in symbiotic cells of *B. diazoefficiens* (22).

We surveyed all genes that were down-regulated in the Δ*nasT* mutant (Table 2) for possible NasT-interaction hairpin formation within their mRNA leader regions (39). Among the genes examined, only two exhibited a clear NasT-interaction hairpin within the leader region: one was a putative *coxS* gene (blr0335; Fig. S1) and the other was *nirA* (bll4571; Fig. S1), encoding the assimilatory NO_2_^−^ reductase. Thus, it is reasonable to expect a direct interaction between NasT and the hairpin within their leader regions (Fig. S1). However, the effects of NasT on the expression of the other genes may have been indirect; alternatively, NasT-binding motifs in these genes may not be well conserved and NasT may regulate these genes by an alternative mechanism. Nevertheless, we cannot rule out the possibility that some of these genes are regulated by unknown mechanisms that differ to NasT in response to the phenotype promoted by the *nasT* deletion.

### Reduced expression of genes involved in nitrogen acquisition in the Δ*nasT* mutant is counteracted by the induction of genes with similar functions

**Fig. 2:**
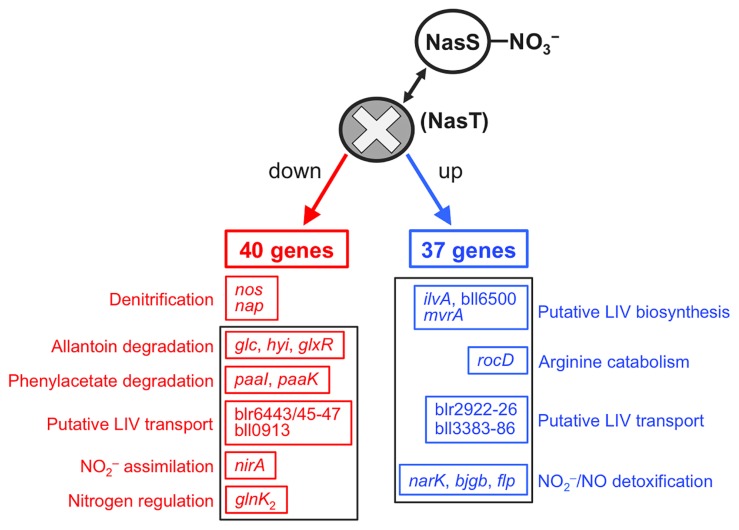
Summary of transcription analysis results in the Δ*nasT* mutant under denitrifying conditions. See the text for details.

The expression of a number of genes related to nitrogen acquisition, such as *glc*-*hyi*-*glxR* (allantoin degradation), *paaIK* (phenylacetate degradation), blr6443/45-6447 and bll0913 (LIV transport), *nirA* (assimilatory NO_2_^−^ reductase), and *glnK*_2_ (nitrogen regulatory protein PII), was down-regulated (Table 2 and Fig. 2) (1, 6, 13, 21, 36). The PII protein encoded by *glnB* was also down-regulated in the Δ*nasT* mutant of *Paracoccus denitrificans* (17).

In contrast, other genes related to the transport, synthesis, and catabolism of amino acids were up-regulated in the absence of NasT (Table 3 and Fig. 2). Among them, we found two putative systems for LIV transport (blr2922-2926 and bll3383-3386); genes putatively involved in LIV synthesis: bll6500, bll6502 (threonine dehydratase *ilvA*), and *mvrA* (ferredoxin NADP^+^ reductase that may provide low-potential electrons for amino acid synthesis); and *rocD* encoding ornithine aminotransferase for arginine catabolism (7, 12, 13, 23). This result suggests the induction of alternative mechanisms to obtain nitrogen, counteracting the loss of other genes involved in nitrogen acquisition in the Δ*nasT* mutant.

### *narK-bjgb-flp* genes are up-regulated in the Δ*nasT* mutant

Although NasT-recognizable hairpin formation was predicted in the leader regions of *narK* and *bjgb* mRNAs (Fig. S1), the expression of *narK*-*bjgb*-*flp* genes was stronger in the Δ*nasT* mutant than in the wild type (Fig. 1 and Table 3). Furthermore, the expression of the *nasC* gene, which is located downstream of *flp*, as part of the same operon (1, 29), remained unchanged in the Δ*nasT* mutant (Fig. 1). The *narK*-*bjgb*-*flp*-*nasC* operon encodes an integrated cytoplasmic system for NO_3_^−^ assimilation and NO_2_^−^/NO detoxification in *B. diazoefficiens* (1, 29). NarK is an MFS (major facilitator superfamily)-type NO_3_^−^/NO_2_^−^ transporter that lowers cytoplasmic NO_2_^−^ levels by exporting NO_2_^−^ to the periplasm, Bjgb is a single-domain hemoglobin that detoxifies NO in the cytoplasm, and Flp is a flavoprotein that functions as an electron donor to the hemoglobin Bjgb and assimilatory NO_3_^−^ reductase NasC (1, 29).

In contrast, Cabrera *et al*. reported that the expression of *narK* was down-regulated in the absence of NasT. Therefore, they suggested the down-regulation of the *narK*-*bjgb*-*flp-nasC* operon in the Δ*nasT* mutant (1). A possible explanation for this discrepancy is that the researchers employed aerobic NO_3_^−^-assimilation conditions (*i.e*., NO_3_^−^ as the sole nitrogen source). However, we herein employed denitrifying non-assimilation conditions; under anaerobiosis with NO_3_^−^ as the electron acceptor and ammonia and organic nitrogen as nitrogen sources (2, 26). The present results suggest that the function of *narK*-*bjgb*-*flp*-*nasC* genes is important beyond NO_3_^−^-assimilation conditions and may be subjected to a complex regulatory system. In support of this hypothesis, RegR, the response regulator of the RegSR two-component regulatory system, has been shown to activate the transcription of the *narK*-*bjgb*-*flp*-*nasC* operon under denitrifying conditions, with a putative RegR box located upstream of *narK* (37). Additionally, we found a putative FixK box upstream of *bjgb* (data not shown), suggesting the control of a FixK-like transcriptional regulator in response to low oxygen conditions (14, 19).

Notably, the expression of *nasC* (encoding the assimilatory NO_3_^−^ reductase) and *nirA* (encoding the assimilatory NO_2_^−^ reductase) differed with respect to their dependence on NasT (Table 2 and 3). Under our experimental conditions, the *nirA* expression pattern was similar to that reported by Cabrera *et al*., in which *nirA* was down-regulated in the Δ*nasT* mutant (1). In contrast to the majority of bacteria in which the genes encoding an assimilatory NO_3_^−^ reductase or NO_2_^−^ reductase are arranged in the same operon (15), *nirA* in *B. diazoefficiens* is located downstream of NasST, while *nasC* is located at a separate locus (30). The separate location of *nasC* supports *narK*-*bjgb*-*flp*-*nasC* genes being subjected to NasT-independent regulation, as observed under the experimental conditions employed in the present study.

### Periplasmic nitrate reductase is responsible for anaerobic nitrate reduction in the Δ*nasT* mutant

**Fig. 3:**
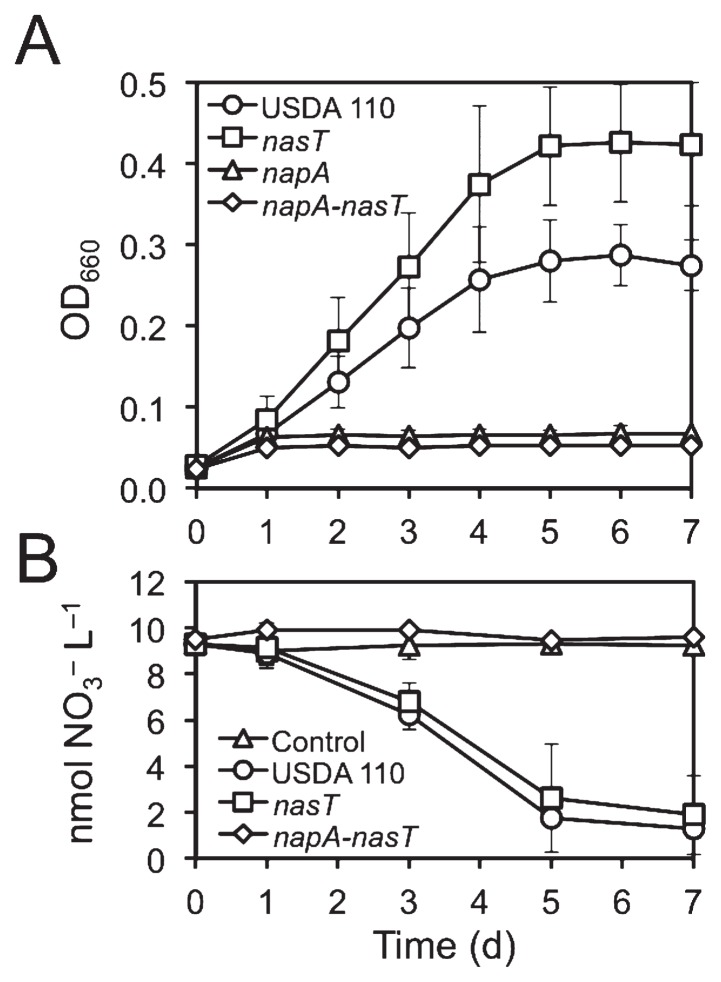
Growth of *B. diazoefficiens* under denitrifying conditions in HMMN medium. (A) Growth of wild-type USDA 110 and the indicated mutant strains. Growth was measured by recording optical density at 660 nm on a daily basis. (B) Extracellular concentrations of nitrate (NO_3_^−^) are indicated for the cultures shown in (A). The results presented are the means of at least three biological replicates±standard deviations (*n*=3–5).

An interesting finding from a previous study showed that although *nap* expression and Nap activity both markedly decreased in the Δ*nasT* mutant under denitrifying conditions, growth was more rapid than that of the wild type (30). The enhanced growth of the Δ*nasT* mutant under denitrifying conditions was confirmed in the present study (Fig. 3A). Consistent with previous findings by Delgado *et al.* (4), the growth of the Δ*napA* single mutant was completely abolished (Fig. 3A). The growth of the Δ*napA*-Δ*nasT* double mutant was also abolished (Fig. 3A), indicating that Nap is the sole enzyme responsible for reducing NO_3_^−^ to NO_2_^−^ under anaerobic NO_3_^−^-respiring conditions. The Δ*nasT* mutant and wild-type strain consumed NO_3_^−^ at a similar rate from the growth medium, but the consumption was completely abolished in the Δ*napA*-Δ*nasT* double mutant (Fig. 3B). These results indicate that a reduced level of Nap is still sufficient to sustain the anaerobic NO_3_^−^ reduction in the Δ*nasT* mutant.

### Involvement of nas genes in the growth enhancement of the Δ*nasT* mutant

**Fig. 4:**
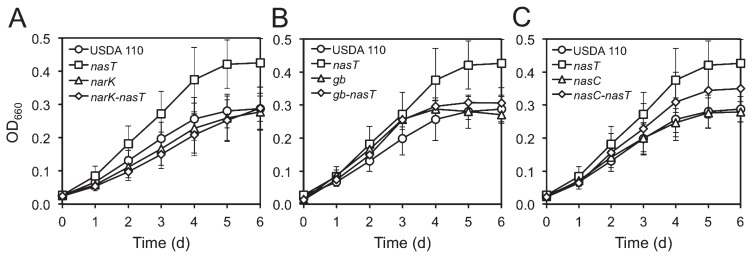
Involvement of *narK*, *bjgb*, and *nasC* genes in the growth of *B. diazoefficiens* under denitrifying conditions in HMMN medium. (A) Growth of Δ*narK* and Δ*nasT*-Δ*narK* mutants. (B) Growth of Δ*bjgb* and Δ*nasT*-Δ*bjgb* mutants. (C) Growth of Δ*nasC* and Δ*nasT*-Δ*nasC* mutants. Wild-type USDA 110 and the Δ*nasT* mutant are shown as a reference in all charts. Growth was measured by recording optical density at 660 nm on a daily basis. Results presented are the mean of at least three biological replicates±standard deviations (*n*=3–5).

**Fig. 5:**
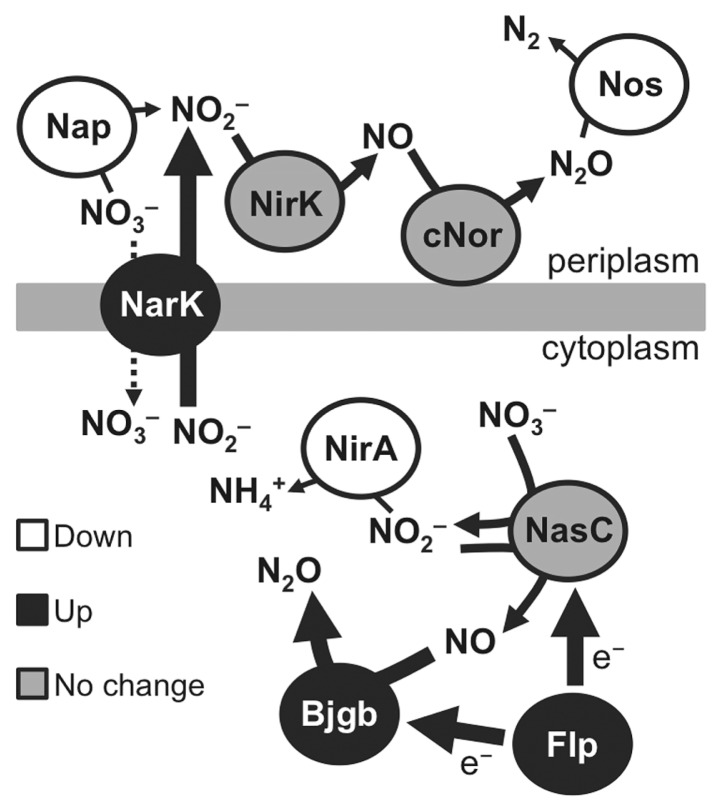
Model of the function of denitrification genes, the *nas* gene cluster, and *nirA* products in the Δ*nasT* mutant. Proteins in white, black, or gray indicate the down-regulation, up-regulation, or unchanged regulation of the respective genes in the Δ*nasT* mutant. See the text for details.

We investigated whether the *narK*-*bjgb*-*flp*-*nasC* operon is involved in the growth enhancement of the Δ*nasT* mutant under denitrifying conditions. The additional loss of the *narK* or *bjgb* genes in the Δ*nasT* mutant background suppressed the growth enhancement of the Δ*nasT* single mutant (Fig. 4A and B). This result indicates that the *narK* and *bjgb* genes are necessary for the enhanced growth of the Δ*nasT* mutant. The mechanisms responsible for this enhancement may be related to the increased capacity of the Δ*nasT* mutant to detoxify NO_2_^−^ and NO in the cytoplasm (1) (Fig. 5). NarK may act to reduce cytoplasmic NO_2_^−^ levels, which are presumably the result of the decreased expression of *nirA* (Table 2 and Fig. 5); Bjgb-Flp may reduce NO, which is produced by NasC during anaerobic nitrate-dependent growth, as reported previously (1).

The additional loss of *nasC* in the Δ*nasT* background resulted in an intermediate growth phenotype between the Δ*nasT* single mutant and wild type (Fig. 4C). This result suggests that the assimilatory NO_3_^−^ reductase NasC is active in the Δ*nasT* mutant and is involved in the growth enhancement of the Δ*nasT* mutant. The contribution of NasC to energy production may be related to its capacity to reduce NO_2_^−^ to NO (1) (Fig. 5).

## Conclusions

Our results suggest the following: (i) NasT is a key regulator for genes associated with nitrogen metabolism under denitrifying conditions, particularly for branched-chain amino acid transport; (ii) the direct NasT regulatory mechanism that was described for *nos* genes (31) may not be common for other targets because most of them did not exhibit a NasT-interaction hairpin; and (iii) the transcription of some NasT targets may be enhanced in a NasT-independent manner under non-assimilation denitrifying conditions, as observed for the *narK*-*bjgb*-*flp*-*nasC* operon.

According to the model proposed by Cabrera *et al.* (1), an explanation for the events that occur in the *B. diazoefficiens* Δ*nasT* mutant under denitrifying conditions is as follows (Fig. 5). The loss of genes in the Δ*nasT* mutant may induce genes responsible for alternative nitrogen acquisition, including *narK*-*bjgb*-*flp*-*nasC* (Fig. 2). As a consequence of the induction of the *narK*-*bjgb*-*flp*-*nasC* operon, the growth of *B. diazoefficiens* under denitrifying conditions is induced, which may be explained by the enhancement of NO_2_^−^ and NO detoxification systems in the cytoplasm (1) (Fig. 5).

These results may provide a novel approach for enhancing the denitrifying growth of *B. diazoefficiens* and other bradyrhizobial strains by optimizing the NO_2_^−^ and NO detoxification systems. This may have important implications for improving the survival of bradyrhizobial cells in the soybean rhizosphere and for root colonization, as well as for the modulation of NO levels in soybean nodules and N_2_O levels in soybean fields (9, 11, 18, 27).

## Supplemental materials



## Figures and Tables

**Table 1:** Bacterial strains and plasmids used in the present study.

Strain/plasmid	Relevant characteristics	Source/reference
Strains		
*Bradyrhizobium diazoefficiens*		
USDA 110	Wild type	13
Δ*nasT*	*nasT*^−^; *nasT*::del	30
Δ*napA*	*napA*^−^; *napA*::Ωcassette; Sp^r^ Sm^r^	9
Δ*nasC*	*nasC*^−^; *nasC*::del	This study
Δ*narK*	*narK*^−^; *narK*::del	This study
Δ*bjgb*	*bjgb*^−^; *bjgb*::del	This study
Δ*napA*-Δ*nasT*	*napA*^−^* nasT*^−^; *napA*::Ωcassette, *nasT*::del; Sp^r^ Sm^r^	This study
Δ*nasC*-Δ*nasT*	*nasC*^−^* nasT*^−^; *nasC*::del, *nasT*::del	This study
Δ*narK*-Δ*nasT*	*narK*^−^* nasT*^−^; *narK*::del, *nasT*::del	This study
Δ*bjgb*-Δ*nasT*	*bjgb*^−^* nasT*^−^; *bjgb*::del, *nasT*::del	This study
*Escherichia coli*		
DH5α	*recA*; cloning strain	Toyobo
Plasmids		
pRK2013	ColE1 replicon carrying RK2 transfer genes; Km^r^	5
pK18*mobsacB*	Suicide vector; Km^r^	32
pΔ*nasT*	pK18*mobsacB*::Δ*nasT*; Km^r^	30
pΔ*nasC*	pK18*mobsacB*::Δ*nasC*; Km^r^	This study
pΔ*narK*	pK18*mobsacB*::Δ*narK*; Km^r^	This study
pΔ*bjgb*	pK18*mobsacB*::Δ*bjgb*; Km^r^	This study

**Table 2:** List of down-regulated genes in the *B. diazoefficiens* Δ*nasT* mutant under denitrifying conditions.

Gene ID^a^	Gene product description	Fold change^b^
bll2540	*nadC*; nicotinate-mononucleotide pyrophosphorylase	−3.08
bll2541	*nadB*; L-aspartate oxidase	−3.25
bll2542	*nadA*; quinolinate synthetase A	−2.47
blr7036	*napE*; periplasmic nitrate reductase protein	−6.8
blr7037	*napD*; periplasmic nitrate reductase chaperone	−5.01
blr7038	*napA*; periplasmic nitrate reductase large subunit	−6.95
blr7039	*napB*; periplasmic nitrate reductase small subunit	−7.73
blr7040	*napC*; membrane-anchored cytochrome *c*	−7.81
blr0314	*nosR*; transmembrane expression regulator/flavoprotein	−3.8
blr0315	*nosZ*; nitrous oxide reductase	−8.99
blr0316	*nosD*; periplasmic protein	−9.29
blr0317	*nosF*; cytoplasmic ABC transporter	−8.53
blr0318	*nosY*; transmembrane permease	−8.78
blr0319	*nosL*; periplasmic copper-binding lipoprotein	−9.12
blr0320	*nosX*; periplasmic flavoprotein	−7.43
blr0321	No similarity	−3.72
blr2896	*paaI*; phenylacetic acid degradation protein	−2.01
blr2897	*paaK*; phenylacetate-coenzyme A ligase	−2.15
bll3150	Putative oxalate:formate antiporter	−2.02
blr6246	ABC transporter substrate-binding protein; putative NitT/TauT family transport system	−2.02
blr6443	ABC transporter permease protein; putative branched-chain amino acid (LIV) transport	−2.46
blr6445	ABC transporter ATP-binding protein; putative LIV transport	−2.45
blr6446	ABC transporter substrate-binding protein; putative LIV transport	−2.64
blr6447	ABC transporter ATP-binding protein; putative LIV transport	−2.03
bll0913	ABC transporter substrate-binding protein; putative LIV transport	−2.07
blr7064	Putative ABC transporter substrate-binding protein	−2.07
blr0335	Putative carbon monoxide dehydrogenase small chain (*coxS*)	−2.02
blr0336	Putative carbon monoxide dehydrogenase large chain (*coxL*)	−2.02
blr3166	*gcl*; glyoxylate carboligase	−5.72
blr3167	*hyi*; hydroxypyruvate isomerase	−6.31
blr3168	*glxR*; oxidoreductase; putative tartronate semialdehyde reductase	−5.09
bll0332	Cytochrome-*c* like protein	−2.94
bll0333	Putative alcohol dehydrogenase	−3.07
bll7610	Conserved hypothetical protein	−2.31
blr2827	Conserved hypothetical protein	−2.29
blr3159	Conserved hypothetical protein	−2.02
blr6840	Conserved hypothetical protein	−2.52
bsr2315	Conserved hypothetical protein	−2.31
bll4571	*nirA*; assimilatory nitrite reductase	−3.14^c^
blr0612	*glnK*_2_; nitrogen regulatory protein PII	−2.05^c^

a An underlined gene identifier indicates the presence of putative NasT-interaction hairpins in the leader region.

b Fold change ≤2; *q* value ≤0.05, unless marked with “^c^”

c *P* value ≤0.05.

**Table 3:** List of up-regulated genes in the *B. diazoefficiens* Δ*nasT* mutant under denitrifying conditions.

Gene ID^a^	Gene product description	Fold change^b^
blr1311	Outer membrane protein	+2.31
blr5221	*hspF*; small heat shock protein	+4.43
blr2806	*narK*; nitrite extrusion protein	+4.86
blr2807	*bjgb*; single domain hemoglobin	+3.22
blr2808	*flp*; FAD-binding protein	+2.31
bll3383	ABC transporter permease protein; putative branched-chain amino acid (LIV) transport	+4.50
bll3384	ABC transporter ATP-binding protein; putative LIV transport	+4.89
bll3385	ABC transporter ATP-binding protein; putative LIV transport	+5.42
bll3386	AraC family transcriptional regulator	+2.30
blr2921	Conserved hypothetical protein	+31.63
blr2922	ABC transporter amino acid-binding protein; putative LIV transport	+18.76
blr2923	Amino acid ABC transporter permease protein; putative LIV transport	+25.39
blr2924	Amino acid ABC transporter permease protein; putative LIV transport	+24.71
blr2925	Amino acid ABC transporter ATP-binding protein; putative LIV transport	+22.85
blr2926	Amino acid ABC transporter ATP-binding protein; putative LIV transport	+24.49
blr6921	Putative multidrug resistance protein	+9.35
bll3369	Putative gluconolactonase	+3.95
bll3376	Oxidoreductase; putative aerobic carbon monoxide dehydrogenase small subunit (*coxS*)	+5.04
bll3377	Oxidoreductase; putative aerobic carbon monoxide dehydrogenase medium subunit (*coxM*)	+4.74
bll6500	Putative SAM (S-adenosyl-L-methionine)-dependent methyltransferase	+2.26
bll6502	Putative threonine dehydratase (*ilvA*)	+2.03
blr3831	*mvrA*; ferredoxin NADP^+^ reductase	+4.24
bll2855	*rocD*; ornithine aminotransferase	+2.18
bll3993	Conserved hypothetical protein	+2.09
bll3994	Conserved hypothetical protein	+2.48
bll6920	Conserved hypothetical protein	+5.42
blr3995	Conserved hypothetical protein	+2.50
blr4566	Conserved hypothetical protein	+6.29
blr4567	Conserved hypothetical protein	+6.19
blr4568	Conserved hypothetical protein	+2.67
bll4091	No similarity	+5.41
bll6133	No similarity	+2.13
bll6134	No similarity	+3.33
blr4022	No similarity	+5.35
blr4764	No similarity	+6.04
blr6135	Putative repressor LexA	+3.24
blr6136	No similarity	+2.96

a An underlined gene identifier indicates the presence of putative NasT-interaction hairpins in the leader region.

b Fold change ≥2; *q* value ≤0.05.
