# Supplementary material for: Identification of Genes Regulated by the Antitermination Factor NasT during Denitrification in Bradyrhizobium diazoefficiens
Source: Microbes Environ. 2019 Jun 28;34(3):260–7. doi: 10.1264/jsme2.ME19033 (PMC6759348; doi:10.1264/jsme2.ME19033)
Supplement: Supplementary file 1 [file 34_260_s1.pdf]

Supplemental materials

Identification of genes regulated by the antitermination factor NasT during denitrification in *Bradyrhizobium diazoefficiens*

Cristina Sánchez*, Arthur Fernandes Siqueira, Hisayuki Mitsui and Kiwamu Minamisawa

Graduate School of Life Sciences, Tohoku University, 2-1-1 Katahira, Aoba-ku, Sendai 980-8577, Japan

Running head: NasT regulon during denitrification

* Corresponding author:
E-mail: cristina.sago@gmail.com; Tel: +34–697–821–861; Fax: +81–22–217–5684

Table S1. Summary of the RNA sequencing result of *B. diazoefficiens* USDA 110 wild-type and Δ*nasT* mutant strains using the Illumina HiSeq 2000 Sequencing System.

Sample name	Yield (Mbases)	No. of reads	% ≥ Q30 bases	Mean quality score	No. of reads after trimming	% of mapped reads
WT_1	4,640	45,935,660	83.8	33.5	39,569,298	44.2
Δ*nasT*_1	4,588	45,422,248	83.8	33.5	38,912,970	40.3
WT_2	4,537	44,916,880	82.2	33.2	37,211,938	36.8
Δ*nasT*_2	4,483	44,388,194	82.9	33.3	37,845,918	46.8

Table S2. Primers defined in this study.

Name	Sequence (5'→3')	Reference
*sigA*f	GAGAACCAGATGTCGCTTGC	(1)
*sigA*r	TGGATGTCCTGCTCCTGAAG	(1)
napAf	ACCAAGGTGGTCTCGTTCTG	(2)
napAr	TCTTTCCGGTCAGAAGATGG	(2)
*nirK*f	ATGGCGACTATGTCTGGGAG	(2)
*nirK*r	TGAACTTGTACATCGCAGCC	(2)
*norB*f	ATCTACGTCCTGCCCAACAC	(2)
*norB*r	AGTAGGTCGCACCCATGAAG	(2)
*nosZ*f	TCCGACTACCAAGGCAAATAC	(1)
*nosZ*r	TCCGCTTGAGGTTGAAGATG	(1)
*nosR*f	CCGCTGATTTTCCTTCTCTGG	(2)
*nosR*r	TTGGCGGCGTTGTTGAG	(2)
*narK*f	CTGGATCATGGCGTACCTCT	This study
*narK*r	GACGGAAACTGCGTCTTGAT	This study
*nasC*f	CTATGTCGCCAACAAGCTGA	This study
*nasC*r	GAGCCGACGAAGACCAGTAG	This study
bll3385f	GTGGCTGATCTTCGCAGTCT	This study
bll3385r	CTCGCGACGAGGAAGTAGAG	This study
*nasC*_01	GGAATTCCTCAATGCCAGCACGAAGT[a]	This study
*nasC*_02	CGCGATCAGGCGCTTCAGCTGGCAGGTGGTCTTGGTGGTC	This study
*nasC*_03	GACCACCAAGACCACCTGCCAGCTGAAGCGCCTGATCGCG	This study
*nasC*_04	CGGGATCCGCCAATGCAGAAGATAGCAG[b]	This study
*narK*_01	CGGGATCCTTCTGATGATCACCCACGAC[b]	This study
*narK*_02	GACGCAGGTAGAACCACCAGGCGATAGTCTTGCCAGTCGC	This study
*narK*_03	GCGACTGGCAAGACTATCGCCTGGTGGTTCTACCTGCGTC	This study
*narK*_04	CCCAAGCTTCATACCGTTACCGACGATGA[c]	This study
*bjgb*_01	CGGGATCCTCTATTTCGTCGGGCAGAAG[b]	This study
*bjgb*_02	CTCGCCGAGACCCTTCTCCACCGCGGCGGTCTCCGAAATC	This study
*bjgb*_03	GATTTCGGAGACCGCCGCGGTGGAGAAGGGTCTCGGCGAG	This study
*bjgb*_04	CCCAAGCTTGACCAGCGTCTTGAGGAGAT[c]	This study
*nasT*_01	TTTGCATTCGTTGTCTCAGG	This study
*nasT*_02	CCAGAACCGCAATTGGTAAT	This study

Restriction enzyme recognition sites are underlined: [a]EcoRI; [b]BamHI; [c]HindIII.

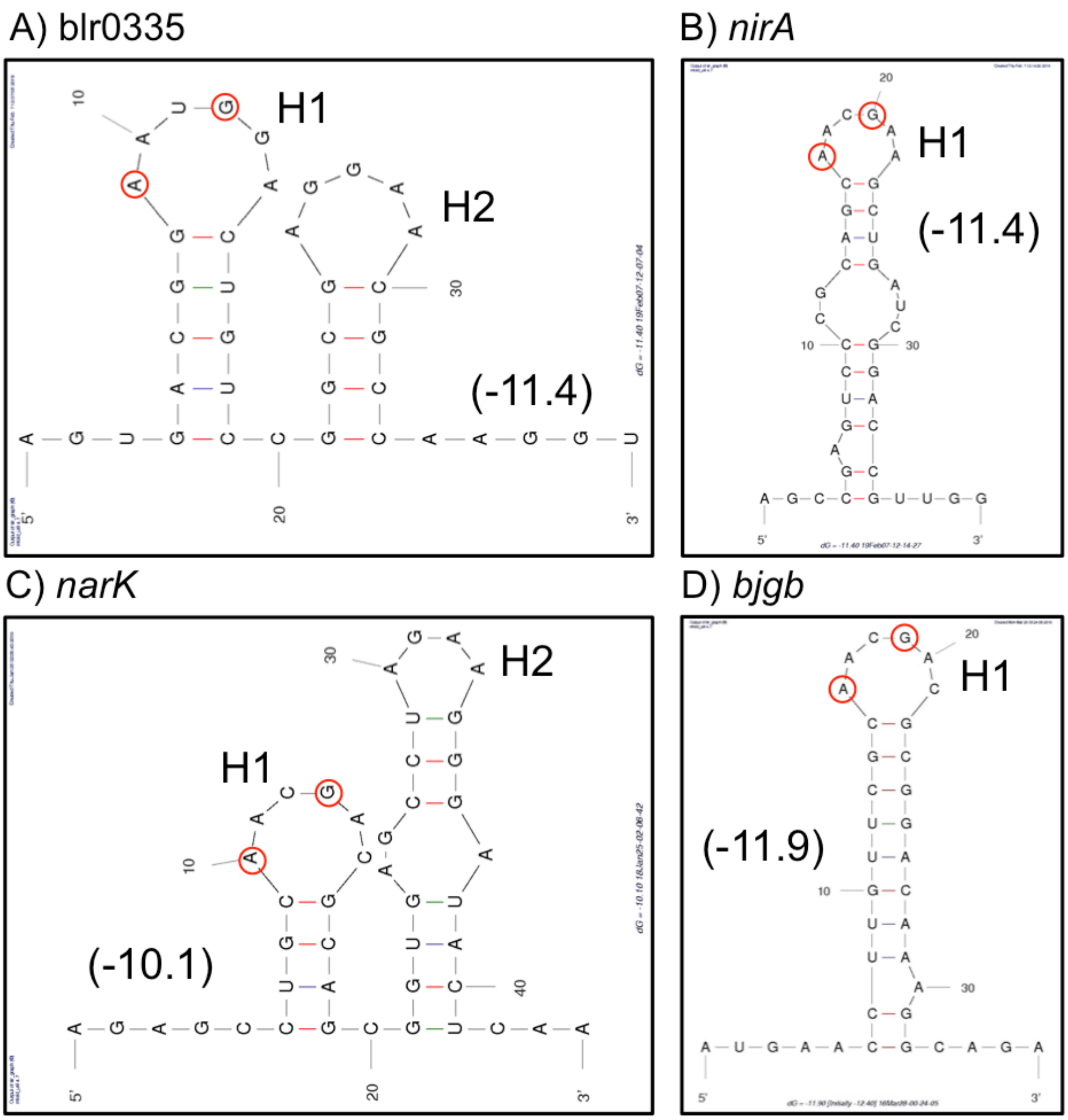

Figure S1. Predicted hairpins by Mfold (3) within blr0335, *nirA*, *narK*, and *bjgb* leader RNAs of *B. diazoefficiens*. Conserved A and G within the terminal loops of H1 hairpin are inside red circles. Values of the change in free energy (kcal mol^{-1}) are indicated in parenthesis.

REFERENCES

1. Itakura, M., Y. Uchida, H. Akiyama, *et al.* 2013. Mitigation of nitrous oxide emissions from soils by *Bradyrhizobium japonicum* inoculation. Nat. Clim. Change 3:208-212.

2. Sánchez, C., M. Itakura, H. Mitsui, and K. Minamisawa. 2013. Linked expressions of *nap* and *nos* genes in a *Bradyrhizobium japonicum* mutant with increased N_2O reductase activity. Appl. Environ. Microbiol. 79:4178-4180.

3. Zuker, M. 2003. Mfold web server for nucleic acid folding and hybridization prediction. Nucleic Acids Res. 31:3406-3415.
